# Effects of Dietary Phytoestrogens on Hormones throughout a Human Lifespan: A Review

**DOI:** 10.3390/nu12082456

**Published:** 2020-08-15

**Authors:** Inés Domínguez-López, Maria Yago-Aragón, Albert Salas-Huetos, Anna Tresserra-Rimbau, Sara Hurtado-Barroso

**Affiliations:** 1 Department of Nutrition, Food Science and Gastronomy, XaRTA, INSA, School of Pharmacy and Food Sciences, University of Barcelona, 08028 Barcelona, Spain; idominlo8@alumnes.ub.edu (I.D.-L.); myagoara7@alumnes.ub.edu (M.Y.-A.); sara.hurtado_17@ub.edu (S.H.-B.); 2 Andrology and IVF Laboratory, Division of Urology, Department of Surgery, University of Utah School of Medicine, Salt Lake City, UT 84108, USA; albert.salas@utah.edu; 3 Centro de Investigación Biomédica en Red Fisiopatología de la Obesidad y la Nutrición (CIBEROBN), Instituto de Salud Carlos III, 28029 Madrid, Spain; 4 Unitat de Nutrició, Departament de Bioquímica i Biotecnologia, Universitat Rovira i Virgili, 43204 Reus, Spain; 5 Institut d’Investigació Sanitària Pere Virgili (IISPV), 43201 Reus, Spain

**Keywords:** isoflavones, soy, dietary flavonoids, lignans, flaxseeds, endocrine, stages of life, estrogenic, polyphenols, health

## Abstract

Dietary phytoestrogens are bioactive compounds with estrogenic activity. With the growing popularity of plant-based diets, the intake of phytoestrogen-rich legumes (especially soy) and legume-derived foods has increased. Evidence from preclinical studies suggests these compounds may have an effect on hormones and health, although the results of human trials are unclear. The effects of dietary phytoestrogens depend on the exposure (phytoestrogen type, matrix, concentration, and bioavailability), ethnicity, hormone levels (related to age, sex, and physiological condition), and health status of the consumer. In this review, we have summarized the results of human studies on dietary phytoestrogens with the aim of assessing the possible hormone-dependent outcomes and health effects of their consumption throughout a lifespan, focusing on pregnancy, childhood, adulthood, and the premenopausal and postmenopausal stages. In pregnant women, an improvement of insulin metabolism has been reported in only one study. Sex hormone alterations have been found in the late stages of childhood, and goitrogenic effects in children with hypothyroidism. In premenopausal and postmenopausal women, the reported impacts on hormones are inconsistent, although beneficial goitrogenic effects and improved glycemic control and cardiovascular risk markers have been described in postmenopausal individuals. In adult men, different authors report goitrogenic effects and a reduction of insulin in non-alcoholic fatty liver patients. Further carefully designed studies are warranted to better elucidate the impact of phytoestrogen consumption on the endocrine system at different life stages.

## 1. Introduction

**Figure 1:**
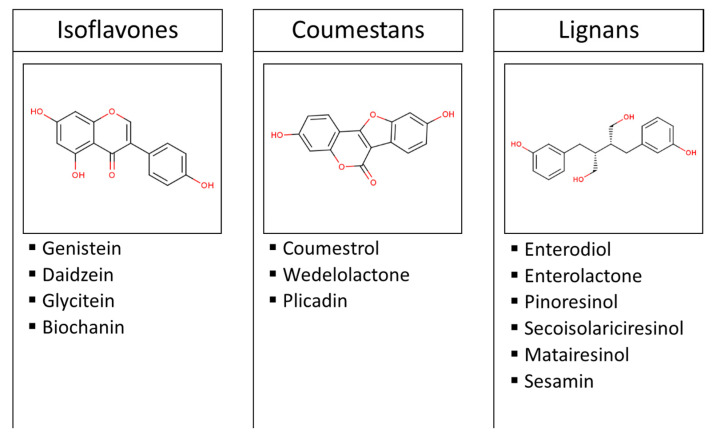
Classification and examples of the most common dietary phytoestrogens. Images are the chemical structures of genistein, coumestrol, and enterodiol.

Phytoestrogens are polyphenolic molecules with a structural similarity to endogenous human hormones, hence their estrogenic activity. The main dietary source of these plant secondary metabolites is legumes (particularly soy), and to a lesser extent fruits, vegetables, and cereals [1]. Figure 1 shows the most common phytoestrogens in diet. According to their origin, lignins are classified into plant lignans (e.g., pinoresinol, secoisolariciresinol, matairesinol, and sesamin) and enterolignans (e.g., enterodiol and enterolactone), which are metabolized from plant lignans by intestinal bacteria [1]. Although ingested in lower quantities than isoflavones and lignans, prenylflavonoids from beer and coumestans from soy are also regarded as polyphenols with estrogenic activity.

The intake of phytoestrogens has increased due to the widespread use of soy products for human consumption and as cattle food [2]. In Europe, the lowest average intake of phytoestrogens occurs in Mediterranean countries, whereas consumption in Northern countries is 0.76 mg/day [3]. The highest soy-derived isoflavone intakes worldwide are still in China and Japan, where the population consumes an average of 15–50 mg per day, compared to only about 2 mg per day in Western countries [4,5]. The promising health effects of soy have driven some people in developed countries to consume it as an alternative to meat or dairy products.

Dietary phytoestrogens are digested in the small intestine, where they are poorly absorbed. Those that reach the liver are conjugated and circulate in the plasma until excretion in urine. Those that are not absorbed are metabolized by the gut microbiota into lower weight compounds [1]. The diversity of food matrices (from pure compounds to complex foods) used in clinical studies could also lead to different results although interindividual variability seems more determinant. It has been demonstrated that phytoestrogen extraction from complex food matrices, such as those with high content of sugars and proteins, is more difficult in in vitro studies; however, no clear differences regarding food matrices were observed in humans [6]. Nevertheless, results using pure compounds must be extrapolated carefully because not only is the matrix different, but also the concentration, which is higher in pure extracts.

**Figure 2:**
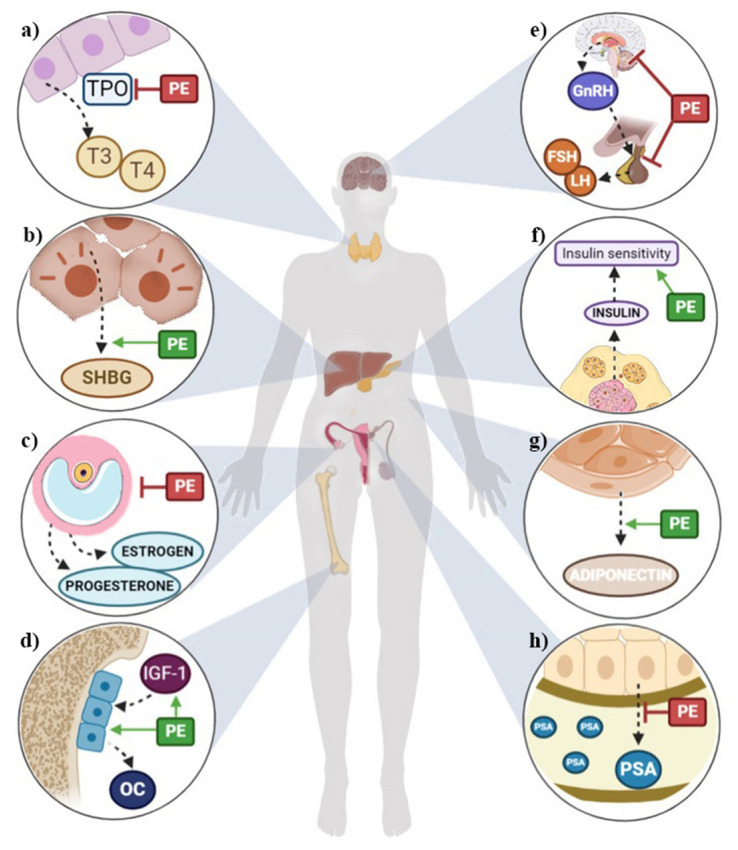
Summary of potential health outcomes of phytoestrogens through the modulation of the endocrine system in (**a**) thyroids, (**b**) liver, (**c**) ovaries, (**d**) bones, (**e**) hypothalamic–pituitary–gonadal axis, (**f**) pancreas, (**g**) fat tissue, (**h**) prostate. FSH: follicle-stimulating hormone; GnRH: gonadotropin-releasing hormone; IGF-1: insulin growth factor 1; LH: luteinizing hormone; OC: osteocalcin; PE: phytoestrogens; PSA: prostate-specific antigen; SHBG: stimulating hormone-binding globulin; T3: triiodothyronine; T4: thyroxine; TPO: thyroperoxidase.

Results from human studies suggest that phytoestrogens may lower the risk of osteoporosis, some cardiometabolic diseases, cognitive dysfunction, breast and prostate cancer, and menopausal symptoms by modulating the endocrine system (Figure 2). However, some authors describe phytoestrogens as endocrine disruptors and believe their beneficial effects have been overestimated [2,5,7]. This ambiguity could be partially due to the variability of published studies, as the beneficial or harmful effects of phytoestrogens depend on the exposure (type, amount consumed, and bioavailability), ethnicity, hormonal status (age and sex and physiological condition), and health status of the consumer [2,5,7].

A plausible mechanism of action for phytoestrogens is estrogen receptor (ER) binding. The effects of isoflavones, which have a five-fold greater affinity for β-ER than α-ER [8], on the endocrine system may be through modulation of the hypothalamic-pituitary axis [9]. However, not all the biological effects of phytoestrogens involve estrogen receptors. They can also activate serotonergic and insulin-like growth factor (IGF) receptors 1, induce free radical binding and modify tyrosine kinases, cycle adenosine monophosphate (cAMP), phosphatidylinositol-3 kinase (PI3K)/Akt, mitogen-activated protein (MAP) kinases, transcription of nuclear factor-kappa β (NF-Kβ), as well as promote DNA methylation and affect histone and RNA expression. In addition, phytoestrogens can act as intracellular regulators of the cell cycle and apoptosis. Thus, due to their antioxidant, antiproliferative, antimutagenic, and antiangiogenic roles, phytoestrogens can improve health [10]. In addition, some authors observed that estrogen and androgen seem to be involved in breast and prostate cancer regulating proliferative and migratory signaling, such as Src/PI3K. Hormonal therapy response may vary depend on interactions between estrogen or androgen receptors and proteins, according to hormone levels [11,12,13].

This integrative review aims to synthesize the results obtained by human studies and assess the potential hormone-related health effects of dietary phytoestrogens throughout the human lifespan.

## 2. Effects of Phytoestrogen Intake on Sex Hormones

The anti-estrogenic activity of phytoestrogens is due to their structural similarity with 17-β-estradiol (E2), the main female sex hormone [5]. As well as interacting with ERs, phytoestrogens can affect the secretion of gonadotropin-releasing hormone (GnRH) [14]. Phytoestrogens could disrupt the endocrine system by interfering with the hypothalamic–pituitary–gonadal axis, which controls estrogen secretion. The hypothalamus releases GnRH and stimulates the pituitary to produce follicle-stimulating hormone (FSH) and luteinizing hormone (LH), gonadotropins that promote the secretion of estrogen, progesterone, and testosterone by the ovaries or testes. Low estrogen levels are a signal for the hypothalamus to release GnRH, whereas high levels provide a negative feedback [15]. Therefore, the presence of exogenous compounds structurally similar to E2 may interfere with this system.

Some studies have focused on how phytoestrogen affects urinary estrogen metabolites, some of which may be involved in the development of breast cancer. In particular, the ratio of 2-hydroxyestrone (2-OH-E1) to 16α-hydroxyestrone (16α-OH-E1) (2:16α-OH-E1) is considered a useful biomarker of estrogen-related cancer risk. A major 2:16α-OH-E1 ratio is related to lower risk of breast cancer. Previously, it was observed that a higher concentration of 16α-OH-E1 was associated with breast and endometrial cancer, while an increase of metabolite 2-OH-E1 seems to inhibit the carcinogenesis [16].

Phytoestrogens have also been reported to affect sex hormones through ER-independent mechanisms of action, such as by altering hormone-binding globulin (SHBG) levels. Circulating estrogens and androgens are mostly bound to albumin and SHBG, with only a small fraction remaining free. As estrogens and androgens are only biologically active in their free form, SHBG affects steroidal activity. In vitro studies have shown that isoflavonoids stimulate the synthesis of SHBG by liver cancer cells [17], but available data from human studies are inconclusive [18,19]. In addition, phytoestrogens inhibit aromatase and other enzymes involved in the synthesis of steroid hormones [20].

Preclinical studies have suggested that phytoestrogens influence sexual function and the incidence of cancer associated with the reproductive system such as ovarian and breast cancer [21], but the results of cross-sectional studies and clinical trials are conflicting [22,23]. In addition to factors such as dose, type, and bioavailability, the effects of phytoestrogens on sexual function could also depend on the life stage of the consumer, as explained below.

### 2.1. Pregnancy

The results of a longitudinal study that measured E2, estriol (E3), testosterone, and isoflavones in urine and serum from 194 pregnant women weakly support the initial hypothesis that genistein and daidzein would reduce levels of E2 and testosterone at the 10th week of gestation. Additionally, sex hormones quantified in umbilical cord serum were not related to isoflavones (genistein, daidzein, and equol) measured at delivery [24].

### 2.2. Children

Dietary phytoestrogens seem to be transferred from maternal blood to the fetus, but there is no evidence that they alter sex hormones in infants [25,26,27]. Although isoflavone bioavailability in this sensitive period may be higher than in adults [28], no estrogenic effects were observed in infants fed with a soy formula [29,30,31]. Nevertheless, a cross-sectional study carried out in children aged 3–6 years reported an increase of androgens in girls and a decrease of estrogens in boys consuming higher amounts of soy and isoflavones [25]. In a crossover trial conducted in girls aged 8–14 years, the consumption of a high-soy diet for 8 weeks significantly increased dehydroepiandrosterone (DHEA) concentrations but not other sexual hormones. Although the level of all sex hormone metabolites excreted was very low, positive correlations with the intervention were found, being higher for total androgens than for estrogens and pregnanediol [32].

### 2.3. Men

A cross-sectional study in randomly selected Japanese men found a negative association between soy product consumption and E2 serum concentrations, but no link was observed with peripheral concentrations of androgen hormones [33]. In a randomized clinical study in Japanese healthy male volunteers consuming 60 mg per day of soy isoflavones, no changes in serum levels of E2 and total testosterone were observed compared to the baseline at the end of the 3-month intervention. However, serum levels of SHBG increased and free testosterone and dihydrotestosterone decreased [34].

There is weak evidence that phytoestrogens contribute to reducing the risk of prostate cancer (PCa). Several observational studies have found a negative association between the consumption of phytoestrogens (soy and its isoflavones) and the levels of prostate-specific antigen (PSA) in blood [35]. PSA, a protein produced by the prostate gland, is used as a marker to detect PCa, although its levels also increase with benign prostate hypertrophy. In a randomized controlled trial, a reduction in PSA levels was observed in men with PCa after consuming soy isoflavones for a mean of 23 days [36]. However, longer studies (minimum 3 weeks and maximum 12 months) did not find beneficial effects on PSA levels after a soy isoflavone intervention [37,38,39,40], nor were changes in PSA plasma levels observed in PCa patients that consumed rye bran bread for 3 weeks [41].

There is a relationship between sex hormones and the pathogenesis of PCa. High levels of androgens, which promote prostate cell growth, may contribute to the risk of PCa in some men. Some epidemiological studies suggest that low levels of testosterone are associated with a lower risk of PCa [42,43]. A meta-analysis of 32 studies published in 2010 by Hamilton-Reeves et al. reported that the consumption of isoflavones had no significant effect on circulating testosterone or free testosterone levels in men [44], in agreement with other clinical trials evaluating phytoestrogen intake in PCa patients [37,39,40,41]. Nor have effects on dihydrotestosterone been described [36,40]. However, in an open-labeled, non-randomized clinical trial of men with higher levels of PSA, free testosterone was depleted after 12 months of daily consumption of 141 mg of isoflavones in soy milk [38].

Recent studies have pointed to a protective role of estrogens in PCa development and progression, alone or in synergy with androgens [45]. Several studies have focused on the beneficial effect of soy isoflavones, specifically genistein and daidzein, as these components can act as weak estrogens. A 6-month randomized controlled study evaluating the effects of isoflavone on men at high risk of developing advanced PCa found an increase in concentrations of the estrogen hormones estrone (E1), E2, 2-hydroxi-estradiol (2-OH-E2), and 16α-OH-E1. An increase in the 2:16α-OH-E1 ratio was also reported, which is related to a reduced risk of estrogen-mediated cancer. No differences were observed for 2-methoxyestradiol (2-ME2), 1-methoxyestrone (2-ME1), E3 and 2-OH-E1 [46]. Conversely, Bylund A. et al. reported that levels of E2, FSH, and LH in PCa patients remained unaltered after a 3-week rye bran bread intervention [41].

### 2.4. Premenopausal Women

In agreement with the potential anti-estrogenic effect of phytoestrogens, some authors have observed a significant decrease in estrogen levels after the consumption of soy products [18,22,47,48,49]. In a randomized controlled cross-over trial conducted in 12 healthy premenopausal women, those consuming a high-soy diet for three menstrual cycles had lower urinary concentrations of total estrogens (E1, E2, E3), and some metabolites compared to individuals on a low-soy diet [22]. Although significant correlations were obtained between serum levels of unconjugated estrogens, and urinary conjugated and unconjugated estrogen metabolites, the large intra-subject variability in urinary estrogen levels limits its use as a biomarker [50]. Similarly, a fall in the circulating levels of E2 after the consumption of a soy-rich diet has been reported [47,48,49]. In a cross-sectional study, Kapiszewska M. et al. found an association between low salivary E2 concentrations and the intake of black tea (only or plus green tea), catechins, theaflavins, and epigallocatechin gallate (EGCG), being more pronounced in premenopausal women living in urban areas than in those living in rural areas [51]. As well as a decline in estrogens, a significant decrease in progesterone levels after phytoestrogen consumption has been observed [47,48,49,52]. Conversely, other clinical trials and observational studies do not report any modifications of sex hormones attributed to the consumption of dietary soy-isoflavones [14,19,23,53,54,55,56,57,58].

It has been proposed that changes in estrogen levels induced by dietary phytoestrogens could depend on the individual capacity to produce equol [59,60], mainly the S-equol enantiomer, due to its high affinity for β-ER [61]. Accordingly, Duncan et al. reported that premenopausal equol excretors had a lower risk of breast cancer compared to non-excretors [60].

In summary, it is still uncertain if a phytoestrogen-rich diet triggers an imbalance of estrogen and progesterone concentrations. In randomized controlled crossover trials, no significant changes were observed in the progesterone/E2 ratio in women who consumed a soy diet for two menstrual cycles [56], whereas the ratio increased after the intake of 10 g/day of flaxseeds for three menstrual cycles [62].

The status of sex hormones can also be an indicator of breast cancer risk. Although measuring estrogen and progesterone concentrations in nipple aspirate fluid may be better than using serum samples to detect dietary-associated changes in the breast, the correlation between dietary phytoestrogen and estrogens was poor in both matrices [58]. On the other hand, the scant evidence for an increase in the 2:16α-OH-E1 ratio after the consumption of soy and flaxseed suggests such high-phytoestrogen foods may have a protective role against breast cancer [22,48,63]. In the same vein, Xu et al. observed a decrease in the ratio of genotoxic metabolites (16α-OH-E1, 4-hydroxyestradiol (4-OH-E2), and 4-hydroxyestrone (4-OE-E1)) and total estrogens [22]. However, no effects on biomarkers related to breast cancer risk have been reported in other studies [23,56,59].

Inconclusive effects of phytoestrogen supplementation in the form of soy protein powder (low and high doses) on the concentrations of FSH and LH have been observed. Both hormones decreased after low- but not high-isoflavone diets [18], as well as after the consumption of soy products [52,64]. However, other studies found no significant changes in FSH and LH concentration after phytoestrogen supplementation [14,23,48,49,53,54,55].

Most authors have not found any effects of a phytoestrogen-rich diet on circulating levels of androgens [23,53,56,57,64,65]. Two clinical trials did report a decrease of DHEA-sulfate concentrations in healthy premenopausal women after 1 to 3 months on a diet high in soy and soy products [18,47]. In contrast, in a randomized controlled clinical trial (RCT) conducted in healthy premenopausal women consuming 10 g/day of flaxseeds for three menstrual cycles, an increase in serum levels of testosterone in the mid-follicular phase was observed [62]. Overall, there is no solid evidence supporting the influence of phytoestrogens on SHBG [19,23,49,52,53,55,56,57,62,64,65,66], although a weak increase has been described [18,67,68].

A prolonged menstruation after regular intake of phytoestrogens has been reported [62,68], but most studies indicate no significant changes in menstrual cycle length or concentration of prolactin [18,23,53,56,57,62,65,66].

### 2.5. Postmenopausal Women

Menopausal transition is caused by the depletion of ovarian follicles and their responsiveness to the pituitary gonadotropins FSH and LH. This results in low serum levels of the ovarian hormones estrogen and progesterone, and also an increase in FSH concentrations due to the disruption in the negative feedback regulating the hypothalamic–pituitary–gonadal axis [69]. These hormonal changes are responsible for several menopausal symptoms, such as vasomotor symptoms, hot flushes, and vaginal dryness, as well as long-term disorders like osteoporosis, cardiovascular diseases, and breast cancer.

In postmenopausal women there is little evidence supporting the hypothesis that phytoestrogens affect sex hormone levels. Numerous studies have reported that phytoestrogens—including isoflavones, flavonoids, and lignans—do not affect estrogen or progesterone concentrations in postmenopausal women [7,14,19,70,71,72,73,74,75,76,77,78,79,80,81,82,83,84,85,86,87,88,89,90,91,92,93,94,95]. However, other clinical studies did find that isoflavone administration produced significant changes in E2 [96,97] or progesterone concentrations [98]. Two other postmenopausal studies suggested that flaxseed lignans may reduce E2 and E1 sulfate [99] in healthy women, and E1 concentrations in obese and overweight women [100]. Tormala R. et al. (2008) also reported lower E1 concentrations after soy protein supplementation in tibolone-using postmenopausal women who were equol producers [101]. In one epidemiological study, the relationship between isoflavone intake and peripheral E2 concentrations was assessed in postmenopausal women, and urinary excretion of daidzein, genistein, and glycitein and serum levels of daidzein and glycitein were associated with lower plasma E2 levels. Interestingly, these associations were stronger in 18 postmenopausal women with the CC genotype for ESR1 *Pvull* polymorphism, suggesting that genes influence diet effects [102].

Phytoestrogen effects on urinary estrogen metabolites have been studied in order to assess their potential protective role against breast cancer. One of the most recent studies reports an increase in the 2:16α-OH-E1 ratio after red clover-derived isoflavone supplementation in postmenopausal osteopenic women [103]. These results are consistent with prior research that found an increase in the 2:16α-OH-E1 ratio after flaxseed supplementation [104,105]. Other studies, however, did not find any difference in the ratio after phytoestrogen supplementation in postmenopausal women [81,95], and one even reports a lower ratio [106].

Other hormones affected by the disruption of the hypothalamic–pituitary–gonadal axis are the gonadotropins FSH and LH, which according to different clinical trials are not affected by phytoestrogen supplementation [14,79,82,83,107,108,109]. Only Crisafulli A et al. found lower gonadotropin levels after 54 mg/day of genistein supplementation for 6 months in 60 postmenopausal women compared to the control group [110].

Whereas some clinical studies found that isoflavone consumption increased SHBG levels in postmenopausal women [18,71,96,110], others concluded the opposite. Thus, Wu A.H. et al. (2012) and Uesugi S et al. report lower concentrations of SHBG in healthy postmenopausal women after supplementation with EGCG or isoflavones [91,97], but most of the studies found no association between SHBG levels and phytoestrogen intake [19,78,79,81,83,85,86,88,95,99,100,102,108,109,111,112,113]. Lastly, results from epidemiological studies support the hypothesis that some phytoestrogens may have a positive influence on SHBG. Monroe K.R. et al. showed that plasma enterolactone levels were associated with higher concentrations of SHBG in postmenopausal Latina women [114]. Low et al. also reported higher concentrations of SHBG in postmenopausal women with higher urinary excretion of lignans, but no association was found with the excretion of other phytoestrogens, such as isoflavones, equol, or O-desmethylangolesin (O-DMA) [115].

To date, most of the few human studies evaluating phytoestrogen effects on androgens in postmenopausal women have found no associations between phytoestrogen intake and androgen peripheral concentrations [74,82,85,86,91,109,116]. Yet Basaria S. et al. reported a decrease in testosterone levels after 12 weeks of isoflavone supplementation, results that were supported by Kapoor R. et al. only in normal-weight postmenopausal women consuming pomegranate for 3 weeks [111,113]. Bioavailable testosterone remained unchanged in both trials. Furthermore, Wu W.H. et al. also reported lower levels of DHEA-sulphate, an androgen precursor, in postmenopausal women after a 5-week intervention with sesame lignans [81].

## 3. Effect of Phytoestrogen Intake on Thyroid Hormones

It is not clearly established if phytoestrogen consumption alters the hypothalamic–pituitary–thyroid axis and triggers goitrogenic effects in humans [9,117]. A randomized, double-blind, and cross-over trial carried out in 60 patients with subclinical hypothyroidism and an adequate iodine intake reported an advance to overt hypothyroidism in 10% of cases (6 females) after administering soy protein with isoflavones for 8 weeks [118]. Nevertheless, soy isoflavones appear not to affect euthyroid populations with an optimal iodine status [9,117]. Controversial results have been obtained by studies in healthy humans, who did not experience anti-thyroid or any other effects after consuming dietary phytoestrogens, particularly isoflavones. The evidence for the impact of phytoestrogens on thyroid function according to the life stage is provided below.

### 3.1. Pregnant Women

A cross-sectional study found no association between soy consumption in early pregnancy and the development of thyroid dysfunction or autoimmunity in 505 women living in areas with an optimal intake of iodine [119]. However, further studies in countries with iodine deficiency are needed.

### 3.2. Children

A cross-sectional study in children aged 8–15 years in an iodine-deficient region of the Czech Republic did not obtain conclusive results. Although levels of free thyroxine (free-T4) increased after a higher intake of soy, a positive correlation was observed between serum daidzein levels and thyroid stimulating hormone (TSH) [120]. In a retrospective study, children with congenital hypothyroidism fed with soy formula had a higher concentration of TSH compared to those fed with non-soy formula [121]. Nevertheless, a study in 12 hypercholesterolemic children consuming toffee candies containing isoflavone extract for 8 weeks did not find any effect on TSH, triiodothyronine (T3), or T4 levels [122].

### 3.3. Men

Two clinical trials showed that products with a high phytoestrogen content had anti-thyroid effects in male populations [123,124]. Hampl R. et al. observed a significant increase of serum TSH in young males after supplementation with 2 g/kg body weight/day of boiled unprocessed natural soybeans for 7 days [123]. Similarly, significant changes in TSH (increasing) and free-T4 (decreasing) were found in men with type-2 diabetes mellitus and compensated hypogonadism after consumption of 15 g/day of soy protein isoflavones for 3 months [124].

### 3.4. Premenopausal Women

No significant changes in thyroid function were found in healthy and obese premenopausal women consuming soy isoflavones for periods of 1 week to 6 months [53,123,125], but reduced free-T3 levels were found in healthy young females following a high-soy diet for three menstrual cycles [126].

### 3.5. Postmenopausal Women

A randomized, double-blind and parallel trial conducted in 120 postmenopausal women reported a significant increase in TSH and a decrease of free-T4 after the intake of 66 mg/day of soy isoflavones for 6 months [124]. Jayagopal V. et al. obtained similar results in a cross-over design trial involving 32 postmenopausal women with type-2 diabetes mellitus consuming twice the amount for half the duration [109]. Another parallel RCT conducted by Mittal et al. in oophorectomized women showed a decrease in free T3 after a 12-week intervention with 75 mg/day isoflavone [127]. Other authors found no significant differences in the level of thyroid hormones, but other thyroid-related parameters such as thyroxine-binding globulin (TBG) and the T3:T4 ratio were altered, indicating possible goitrogenic activity derived from phytoestrogen consumption [18,108]. In addition, Sosvorová et al. observed the presence of mono-iodinated derivatives of daidzein and genistein in urine after daily consumption of isoflavonoids for 3 months, which could explain the entry of genistein and daidzein in human thyroid follicles and thyroperoxidase modification [108]. However, other RCT observed no significant changes in thyroid function after isoflavone intake [84,128,129,130]. Nor was an effect reported in longer studies (1 to 3 years of duration) after the administration of different doses of isoflavones (2–200 mg/day) [74,93,131,132,133], possibly because adaptation to long-term changes in dietary isoflavone intake triggers endocrine autoregulation [95].

## 4. Effect of Phytoestrogen Intake on Cardiometabolic Risk-Related Hormones

Cardiometabolic diseases encompass a set of dysfunctions affecting the cardiovascular system. These are not limited to hard cardiovascular events such as coronary heart disease, myocardial infarction, and stroke, but also include cardiovascular risk factors, namely obesity, insulin resistance, endothelial dysfunction, atherosclerosis, lipid profile, or non-alcoholic fatty liver disease, among others [134].

Obesity is usually the first risk factor that triggers chronic low-grade inflammation, which plays a crucial role in systemic metabolic dysfunction. Adipose tissue and adipocytes are dysfunctional in obese individuals, causing the secretion of pro-inflammatory adipokines that contribute to chronic inflammation and subsequently to the progression of cardiometabolic disorders like insulin resistance [135,136,137].

Another condition that increases the odds of suffering cardiovascular diseases is type-2 diabetes mellitus, which involves alterations in intestinal sensitivity to insulin and glucagon-like peptide-1 (GLP-1), as does its previous state, insulin resistance. GLP-1 has a variety of metabolic effects, including the glucose-dependent stimulation of insulin secretion, and is also involved in cardiovascular health [138]. Insulin, an anabolic hormone secreted in the pancreas, also regulates carbohydrate metabolism, participates in the storage of free fatty acids in adipose tissue, and enhances protein synthesis, increasing amino acid uptake by tissues [139].

Some authors have studied whether phytoestrogens can decrease the levels of pro-inflammatory adipokines such as leptin and resistin or increase adiponectin, an anti-inflammatory hormone, as well as regulate the secretion of insulin, glucagon, and ghrelin. Ghrelin, a recently discovered hormone related to cardiovascular health, is involved in feeding behavior, energy homeostasis, and carbohydrate metabolism. It therefore participates in body weight maintenance, which is crucial for vascular health [140].

### 4.1. Pregnancy

Only one study has evaluated the relationship between phytoestrogens and cardiovascular health during pregnancy. Shi et al. analyzed the association between urinary concentrations of isoflavonoids and cardiometabolic risk markers using data from 299 pregnant women from the NHANES cohort [141]. Those in the fourth quartile of isoflavones had lower levels of insulin and insulin resistance compared to women in the first quartile. None of the individual isoflavonoids (daidzein, equol, and O-desmethylangolensin) in urine were significantly associated with insulin levels.

### 4.2. Adults

To date, two American cross-sectional studies have examined the relationship between phytoestrogens and cardiovascular-related hormones in healthy adults. In one, only lignan intake seemed to be associated with lower fasting insulin in men [142], whereas the other study reported no significant differences [143]. Ferguson et al. took a different approach, inducing transient endotoxemia in young and healthy volunteers and then analyzing the ability of dietary phytoestrogens to reverse the inflammatory response. They found a significant decrease of insulin sensitivity with the higher intake of isoflavones. The participants were asked to follow a healthy diet but did not receive counseling related to soy food intake. Moreover, similar trends were found in two independent cohorts (MECHE and NHANES) [144].

Six RCTs have evaluated the effect of phytoestrogens on cardiovascular health. The participants in four studies were at high cardiovascular risk [145,146,147,148], in one they were men with increased risk of colorectal cancer [149], and in the other they were healthy men [150]. Interventions included isoflavones [149,150], soy nuts [145], daidzein [146], genistein [148], and S-equol [147] administered for periods ranging from 4 weeks to 6 months. Three of the trials found no significant effects on insulin, leptin, or adiponectin [145,146,147]. However, Amanat et al. report that a daily dose of 250 mg of genistein administered to non-alcoholic fatty liver patients for 8 weeks reduced insulin levels [148]. Maskarinec et al. concluded that men who consumed soy early in life had higher levels of leptin, although no association was observed with soy intake during adulthood [150]. Finally, the results of the trial with subjects at high colorectal cancer risk suggested that isoflavones might reduce the insulin-growth factor but only in equol producers [147].

### 4.3. Postmenopausal Women

Most studies on phytoestrogen intake and cardiometabolic hormones have evaluated insulin and insulin resistance (HOMA-IR) in postmenopausal women. The largest observational study included 301 women from The Netherlands and used food frequency questionnaires to assess dietary isoflavone and lignan intake. Individuals with a high lignan diet had lower blood pressure but no significant associations with insulin were observed [151]. In another cross-sectional study women in the highest quartile of lignan or enterolactone intake had better anthropometric profiles and insulin sensitivity [152].

Among published clinical trials, a research group from Italy has carried out several studies monitoring cardiovascular risk factors in women receiving 54 mg of genistein. After a 6-month intervention in 60 healthy women, a decrease in insulin and insulin resistance was observed [110]. Similar results were obtained after 12 and 24 months of intervention in a related study in 389 osteopenic postmenopausal women, who received the same dose of genistein plus calcium and vitamin D [153], the values remaining consistent after an extra year of follow-up in a sub-cohort [154]. Examining the role of metabolic status, Villa et al. divided the intervention group into normo- and hyperinsulinemic patients and found that genistein improved insulin sensitivity indexes only in in the latter [71]. Similarly, women with metabolic syndrome who consumed 54 mg of genistein had lower levels of fasting insulin and HOMA-IR, and higher levels of adiponectin than the placebo group [155,156]. More recently, a research group from Iran assessed the effectiveness of 108 mg of genistein on different metabolic factors in 54 women with type-2 diabetes mellitus in a 12-week intervention. As in the other studies, genistein reduced insulin sensitivity [157].

Other RCTs have used soy isoflavones instead of genistein, administering daily doses of 40–160 mg, far higher than the phytoestrogen intake reported in observational studies. For example, in one RCT the mean daily isoflavone intake in the highest tertile was 11.4 mg [151] as opposed to a total mean intake of 0.06 mg in an observational study [152]. Most of the trials with isoflavones have been performed in healthy postmenopausal women for durations ranging from 8 weeks to 24 months. Generally, the studied vascular-related hormones are leptin, adiponectin, and insulin, although in few cases, ghrelin and resistin have also been evaluated. Results from these trials are ambiguous. Whereas most report no significant changes in any of the aforementioned hormones [158,159,160], others have found beneficial effects on insulin markers in the treatment group compared to the control [118,161,162,163], or a significant increase in adiponectin peripheral levels [164]. Overall, we can conclude that phytoestrogen therapy did not change hormone levels in obese postmenopausal women [70,165], whereas among diabetic women in a randomized cross-over trial there was a significant decrease in insulin resistance in the soy consumers compared to the placebo group [109].

## 5. Effect of Phytoestrogen Intake on Hormones Related to Stress Response

No significant changes in cortisol were observed in healthy women or in those at cardiometabolic risk after consuming soy isoflavones for 2–6 months [18,23,53,95,126]. However, differences between equol excretors and non-excretors have been described in premenopausal women, levels being lower in those who produce this metabolite [60].

## 6. Effect of Phytoestrogen Intake on Hormones Related to Bone Remodeling

Estrogen plays a key role in bone metabolism, contributing to bone mass acquisition in puberty and helping to maintain normal bone density in adulthood [166]. Given that phytoestrogens are structurally similar to estrogens, they can bind to ERs in bone and exert estrogenic actions [167].

Most studies examining the impact of phytoestrogens on bone health measure osteocalcin (OC), a metabolic regulatory hormone secreted by osteoblasts, as it is a sensitive biomarker for bone formation [168]. Parathyroid hormone (PTH), secreted by the parathyroid glands, plays an important role in calcium and phosphate metabolism. As well as stimulating bone turnover, there is increasing evidence that PTH may also promote bone formation [169].

### 6.1. Children

Early-life exposure to soy protein formula did not produce any change in OC and PTH in a clinical study of 48 children [29]. Even though the available data suggest that phytoestrogen intake does not affect bone-related hormones in early stages of life, more studies are needed to clarify this relationship.

### 6.2. Premenopausal Women

The reported effects of dietary phytoestrogen on bone health in premenopausal women are inconsistent. Kwak H.S. et al. found an increase in serum OC after the administration of 120 mg/day of soy-isoflavones for three menstrual cycles. They also observed that high genistein-excretors in the soy group had higher concentrations of OC, suggesting that individual variation may affect the metabolism and functions of isoflavones [54]. However, previous studies report unaltered OC levels [170,171], indicating a need for more research on the phytoestrogen effects on bone metabolism in premenopausal women.

### 6.3. Postmenopausal Women

After menopause, estrogen concentrations decrease dramatically, triggering a greater risk of osteoporosis [172]. Phytoestrogens might improve bone health due to their estrogenic effects, and it has been hypothesized that they could reduce the risk of osteoporosis. Chiechi L.M. et al. and Scheiber M.D. et al. report an increase in OC concentrations in postmenopausal women who consumed a soy-rich diet for 6 and 3 months, respectively. Although uncorroborated by the majority of studies, these results indicate a stimulation of osteoblast activity and suggest that soy may have beneficial effects on bone health [173,174]. It has been suggested that longer treatments may be necessary to produce any change in bone metabolism, but to date neither shorter nor longer studies have reported any alterations in OC related to phytoestrogen intake [74,75,76,77,96,97,107,170,175,176,177,178,179].

In contrast, beneficial effects on bone metabolism through mechanisms of action not involving OC have been described in healthy postmenopausal women [77,97,175,176,177,178,179]. Lambert M.N.T. et al. demonstrated that red clover-derived isoflavones combined with probiotics attenuated estrogen-deficient bone mineral density loss and improved bone turnover even in postmenopausal women with osteopenia [177]. Moreover, a recent meta-analysis and systematic review of RCT with perimenopausal and postmenopausal women concluded that isoflavones can be effective in preserving bone mineral density and attenuating accelerated bone resorption [103]. A possible explanation for these contrasting results could be that estrogens are predominantly antiresorptive agents, so the beneficial effects of phytoestrogens may arise from decreased bone resorption by osteoclasts rather than increased bone formation by osteoblasts.

Lastly, administration of isoflavones or genistein alone for 1 to 24 months did not alter PTH in postmenopausal women [78,92,180,181,182,183]. Only a cross-sectional study carried out with Chinese women found that postmenopausal women with a high intake of isoflavone had lower serum PTH levels [184].

## 7. Effect of Phytoestrogen Intake on Insulin-Like Growth Factors

Insulin growth factor 1 (IGF-1) is part of the growth hormone (GH)—IGF-1 axis and is mostly produced in the liver in response to GH stimulation. Among many other functions, IGF-1 binds its receptor on osteoblasts and enhances bone formation, so any changes in this hormone will have an impact on bone health [185]. Consequently, some studies have used IGF-1 and its binding proteins IGFBP-1 and IGFBP-3 as bone turnover biomarkers.

In addition, several epidemiological studies have shown that higher levels of IGF-1 are associated with an increased risk of different types of cancer. IGF-1 exerts its actions by binding to the IGF-1 receptor, which is expressed in most tissues of the body and stimulates cell proliferation (Cohen DH 2012). Apart from higher levels of IGF-1, several cancers also overexpress its receptor IGF-1R, which has a negative impact on their progression. IGF-2 also appears to be associated with gastrointestinal and gynecological tumors [186].

It has been hypothesized that phytoestrogens may interfere with the IGF system through their effects on steroid hormone physiology or by disrupting GH and IGF signaling [187]. However, the limited evidence in humans is inconclusive, as studies have found both positive and negative results.

### 7.1. Premenopausal Women

To date, only two RCTs have evaluated phytoestrogen effects on IGF-1 and its binding proteins in premenopausal women. In the first study, groups of 14 women consumed soy protein isolates providing 8 mg (control), 65 mg (low dose), or 130 mg (high dose) of isoflavones daily for three menstrual cycles. The low dose significantly increased IGF-1 concentrations compared to the high dose only in the periovulatory phase of the menstrual cycle, although no value was significantly different compared to the control group. A similar result was obtained with IGFBP-3; its concentrations were increased by the low dose diet compared with the high dose in the early follicular phase, but they did not differ from those of the control group [170]. The other study assessed the effects of red clover-derived isoflavone supplementation on IGF-1, IGFBP-1, and IGFBP-3 and its role in breast cancer prevention. This one-month intervention resulted in a non-significant reduction in IGF-1, but this was likely due to differences in IGF-1 levels at baseline between the placebo and the control group. Interestingly, the IGF status was found to be influenced by the stage of the menstrual cycle [188].

Epidemiological data does not support a phytoestrogen effect on IGF levels either. A Japanese cross-sectional study reported that there was no correlation between soy products and isoflavone intake and serum IGF-1 and IGFBP-3 in 261 premenopausal women [189]. Another observational study in women living in Japan and Hawaii also failed to find an association between tofu intake and IGF-1, IGFBP-3, and IGF-1 molar ratio in premenopausal women [190].

### 7.2. Postmenopausal Women

Most clinical studies on IGFs in postmenopausal women have failed to find a protective effect of phytoestrogens against osteoporosis, breast cancer, or colorectal cancer. One of the most recent found no impact on IGF-1 in women with osteopenia after a 24-month intervention [183], which is consistent with other studies reporting that isoflavone supplementation did not alter the IGF system [170,188,191]. Similar results have been obtained for lignan consumption. After administering flaxseed lignans for 3 months, Lucas E.A. et al. found that IGF-I and IGFBP-3 levels were unaltered [88]. An RCT in which 103 postmenopausal women consumed 400 or 800 mg of EGCG for 2 months found no significant changes in IGF-1 or IGFBP-3, although the latter tended to increase in both groups [91].

In contrast with these results, a study comparing the effects of soy protein and milk-based protein reported that both supplements increased IGF-1 levels. Further stratification showed that soy protein had a more pronounced effect on women who were not on hormone replacement therapy [192]. A cross-sectional study found an association between phytoestrogens and growth factors, specifically an inverse association between tofu intake and IGF-1 levels and the molar ratio in postmenopausal women, whereas no changes were observed in those who had never used hormone replacement therapy [190]. However, a similar observational study performed in participants of the Singapore Chinese Health Study did not find any association between soy intake and the IGF-1, IGFBP-3, and IGF molar ratio [193].

### 7.3. Men

It is well established that higher circulating IGF-1 levels are associated with an increased risk of PCa [194], and most studies assessing phytoestrogen effects on adult men have consequently focused on PCa patients. In one of two clinical trials with PCa patients, no changes in IGF-1 or IGFBP-3 were observed after a 3–6-month intervention consisting of 200 mg/day of soy isoflavones [37] and in the other Bylund A. et al. (2003) also found that IGF-1 levels remained unaltered after the administration of rye bran bread for 3 weeks [41]. Conversely, a cross-sectional study with 312 men reported a positive association between soy intake and the IGF-1, IGFBP-3, and IGF molar ratio [193].

## 8. Conclusions

This review has summarized the results of studies on the effects of dietary phytoestrogens on endocrine regulation in humans. Although preclinical studies (in vitro and in animal models) show phytoestrogens to be potentially estrogenic compounds, triggering anti-estrogenic effects in the organism, the results of epidemiological studies are ambiguous.

**Figure 3:**
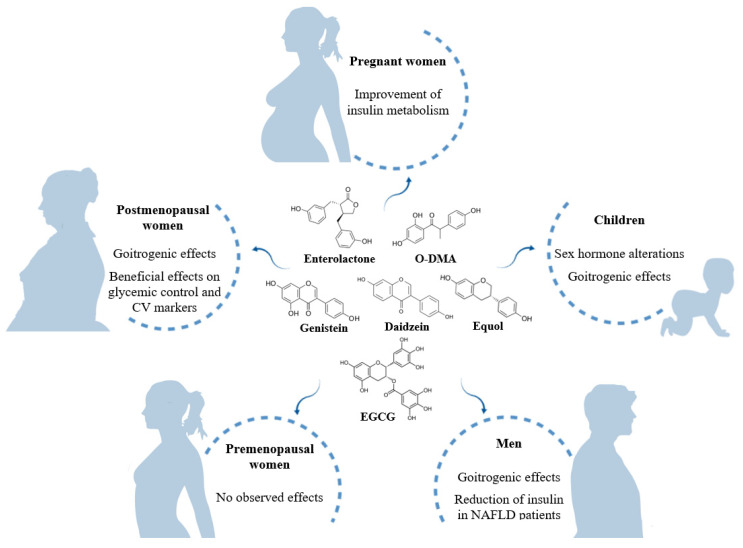
Summary of the effects of dietary phytoestrogens at different life stages. NAFLD: non-alcoholic fatty liver disease.

The impact of phytoestrogens can vary according to the life stage (Figure 3). There is particular concern about how they may affect pregnant women, as this has been poorly studied. Soy isoflavones appear not to have any influence on sex and thyroid hormones, bone remodeling and IGF. However, a study focused on cardiometabolic risk reported a decrease in the level of insulin and insulin resistance in pregnant women consuming higher amounts of isoflavones. Although phytoestrogens transfer from maternal blood to the fetus, no effects have been observed in early life. Nor have endocrine changes been found in infants fed with soy formula, except in a retrospective study carried out in the first year of life of infants with congenital hypothyroidism, which reported an increase of TSH but no conclusive effects on thyroid function. Nevertheless, consumption of phytoestrogens in conditions of insufficient iodine and hypothyroidism may negatively affect thyroid function and favor endocrine imbalance, although such effects have not been observed in euthyroid individuals living in areas with enough supply of iodine. In later stages of childhood, an increase of androgens and decrease of estrogens associated with dietary phytoestrogens have been observed in girls and boys, respectively.

In adulthood, endocrine changes arising from phytoestrogen consumption are unclear, although goitrogenic activity has been observed in men. Effects on sex hormones and IGFs in men are ambiguous, as studies report contradictory results. PCa risk in patients with PCa was unaltered, whereas equol producers with colorectal cancer risk showed a decrease of IGF. Results regarding cardiometabolic risk-related hormones are inconclusive in healthy subjects. Although higher levels of leptin have been reported in early life, no association has been identified in adulthood. However, a reduction in insulin levels was found in patients with non-alcoholic fatty liver.

In premenopausal women, usually studied separately from postmenopausal women, uncertain results have been obtained regarding sex hormones, breast cancer protection, and bone remodeling. Nor has evidence been provided for phytoestrogens affecting IGF levels. Whereas no significant changes in thyroid function were observed, a decrease of free-T3 was found in healthy young females. Among stress response-related hormones, no significant changes in cortisol are described in healthy women or in those at cardiometabolic risk, but a lower production of cortisol is reported in equol-excretors. In postmenopause, the results reported for sex hormones are also ambiguous. However, possible goitrogenic activity derived from phytoestrogen consumption opens up a path for future research. Apart from that, an ameliorative effect has been observed in the cardiometabolic profile of hyperinsulinemic patients, individuals with metabolic syndrome and diabetes. Regarding bone remodeling, the effects of phytoestrogens on OC concentrations are unclear, and their beneficial impact may arise instead from reducing bone resorption by osteoclasts. The results obtained for PHT and IGF are unconvincing, precluding the drawing of any conclusions.

In general, the available evidence for an association between dietary phytoestrogens and endocrine biomarkers is inconclusive. The disparity in results may be due to differences in the type and concentration of the compounds administered and the variety of matrices, which could influence phytoestrogen bioavailability and consequently the effect on hormonal function. Also, while most studies analyze circulating hormones, others report the urinary excretion of metabolites. There is a clear need for further carefully designed studies to elucidate the effects of phytoestrogen consumption on the endocrine system.

## 9. Future Directions

Based on the available literature, we can conclude that intake of phytoestrogens does have some physiological effects in humans related to hormone regulation, but like hormones, the benefits depend on the stage of life. Some factors such as dose and type of compounds, as well as matrices englobing these phytoestroestrogens (food, capsule, etc.) affect their bioavailability and, therefore, the observed results. Most of the research is focused on postmenopausal women and only some have explored the effects during pregnancy and early stages of life. For instance, the effect of phytoestrogen intake on pubertal development has been poorly studied and could lead to interesting results. In order to do that, well-designed intervention trials are key to shed some light on this topic, especially regarding associations that are controversial.
